# Paclitaxel’s Mechanistic and Clinical Effects on Breast Cancer

**DOI:** 10.3390/biom9120789

**Published:** 2019-11-27

**Authors:** Tala M. Abu Samaan, Marek Samec, Alena Liskova, Peter Kubatka, Dietrich Büsselberg

**Affiliations:** 1 Department of Pre-Medical Education, Weill Cornell Medicine-Qatar, Education City, Qatar Foundation, Doha 24144, Qatar; 2 Clinic of Obstetrics and Gynecology, Jessenius Faculty of Medicine, Comenius University in Bratislava, 036 01 Martin, Slovakia; marek.samec@uniba.sk (M.S.);; 3 Department of Medical Biology, Jessenius Faculty of Medicine, Comenius University in Bratislava, 036 01 Martin, Slovakia; peter.kubatka@uniba.sk; 4 Department of Physiology and Biophysics, Weill Cornell Medicine-Qatar, Education City, Qatar Foundation, Doha 24144, Qatar

**Keywords:** breast cancer, anti-cancer therapy, chemotherapy, Paclitaxel, nanomedicine, phytochemicals

## Abstract

Paclitaxel (PTX), the most widely used anticancer drug, is applied for the treatment of various types of malignant diseases. Mechanisms of PTX action represent several ways in which PTX affects cellular processes resulting in programmed cell death. PTX is frequently used as the first-line treatment drug in breast cancer (BC). Unfortunately, the resistance of BC to PTX treatment is a great obstacle in clinical applications and one of the major causes of death associated with treatment failure. Factors contributing to PTX resistance, such as ABC transporters, microRNAs (miRNAs), or mutations in certain genes, along with side effects of PTX including peripheral neuropathy or hypersensitivity associated with the vehicle used to overcome its poor solubility, are responsible for intensive research concerning the use of PTX in preclinical and clinical studies. Novelties such as albumin-bound PTX (nab-PTX) demonstrate a progressive approach leading to higher efficiency and decreased risk of side effects after drug administration. Moreover, PTX nanoparticles for targeted treatment of BC promise a stable and efficient therapeutic intervention. Here, we summarize current research focused on PTX, its evaluations in preclinical research and application clinical practice as well as the perspective of the drug for future implication in BC therapy.

## 1. Introduction

A crucial aspect of the modern era is the rapid progress in the prevalence of many civilization diseases including cancer [1]. Breast cancer (BC )is the most commonly occurring malignant disease in women and the leading cause of cancer death among them and still remains a global problem of public health [2]. Modern approaches in the field of oncology aimed at BC including novelties in diagnosis, treatment, and prevention have a crucial role in the management of cancer. Better knowledge of the biologic heterogeneity of BC leads to the development of more effective therapy concepts in personalized medicine [3]. Over the last decades, substantial progress in the treatment of BC led to the discovery of new drugs with specific actions in cancer suppression. Currently, there are several classes of chemo-therapeuticals based on antimetabolites, alkylating agents, immunological elements, hormonal components, or mitotic deprivation [4]. Recently, two groups of chemotherapeutic drugs (anthracyclines and taxanes) were widely used in adjuvant and neoadjuvant treatment of BC [5]. Paclitaxel (PTX), a class of taxanes, is an antineoplastic drug with an impact on the stabilization of microtubules, which represents a widely used chemotherapeutic agent in numerous cancers. The effect of PTX as an antimitotic drug was documented in a large number of studies [6,7,8]. Moreover, the mechanisms of PTX action associated with the inhibition of tumor growth can act on different levels. In these studies, PTX initiated a cascade of signaling pathways resulting in programmed cell death [9,10]. Modulation of epigenetic markers represents the novelty of cancer-related research, and PTX may also regulate the expression of certain microRNAs (miRNAs) associated with cancer progression. Furthermore, PTX can exert a variety of positive influences on the modulation of immune response via regulation of chemokines, cytokines, or immune cells [11,12]. The resistance of BC to PTX and other chemotherapeutics as a consequence of disequilibrium in various signaling pathways, mutations in certain genes, and epigenetic deregulations is responsible for the worse clinical outcome for patients with BC [13,14,15]. The global challenge in the application of PTX as a dominant anticancer chemotherapeutic agent is the reduction of side effects and increasing drug efficiency. Novelties such as Albumin-bound PTX(nab-PTX) are awesome examples of the progress in the oncology-associated area focused on cross-connection of nanotechnology and cancer treatment [16]. 

In this article, we aimed to summarize the current BC research focused on PTX. The core of our review paper is the conclusion of the most recent data obtained from PTX evaluations in preclinical testing and their application in clinical practice. Finally, we highlight the perspective of the drug within the novel clinical approaches and consequent implications in BC therapy.

## 2. Breast Cancer from the View of Prevalence and Intrinsic Subtypes

BC is the most frequent type of cancer among females, as it constitutes 24% of all female malignancies. BC affects nearly two million females worldwide and is responsible for more than 620,000 deaths annually [2]. Factors such as age, frequency of pregnancies, genetic predisposition, ethnic background, and intake of oral contraceptives all contribute to the increased risk of BC in women [17]. Despite the massive progress in the field of screening tools and programs, the incidence and mortality rates are still rising [18].

Importantly, breast malignancy is a heterogeneous disease characterized by enormous variability in phenotypes and genotypes, meaning that no two patients experience the same clinical features [19]. These differences make the process of targeting BC more complicated. BC can be categorized into 3 main types and 5 subtypes characterized by alterations in the expression of specific genes and the presence or absence of surface receptors resulting in the difference in prognosis and therapy approaches for patients [20]. According to signatures, including the immunohistochemical analysis of receptors, the expression profile of human epidermal growth factor receptor 2 (HER2), and the KI67 proliferative index, these subtypes are classified into HER2 positive (HER2+), luminal types, and triple-negative BC (TNBC) [21]. 

HER2+ BC is the result of the over-expression of the HER2 (ERBB2) gene that encodes a transmembrane glycoprotein receptor p185HER2 [22]. Amplification of HER2 was detected in approximately 15–0% of invasive BC cases. Moreover, a higher frequency of the mutation in HER2 leading to increased expression of the protein was also identified in gastric, esophageal, and other types of cancer [23].

The prevalence of TNBC as the most aggressive form is 10–20%, with higher abundance in the cohort of young women. This molecular subtype is associated with an advanced stage, higher grade of the tumor, overall worse survival rates of patients associated with cancer recurrence, and development of metastasis [24]. Immunohistochemically, TNBC is characterized by the lack of three receptors: estrogen receptor (ER), progesterone receptor (PR), and HER2. Due to the lack of receptors to target, this type of BC is resistant to available treatments [25,26]. Furthermore, TNBC can be classified into claudin-low, basal-like, and molecular apocrine types as a consequence of alterations in gene signatures and histological features (as referred to in Table 1) [27]. Furthermore, hereditary mutations in tumor suppressor genes BRCA1/2 were detected in 15% of patients with diagnosed TNBC [28]. Additionally, recent evidence suggested an association between genes including BARD1, PALB2, and RAD51D and high risk for TNBC [29].

Luminal BC is characterized by the definite presence of ER and the possibility of the presence of PR. Luminal BC can be classified into luminal A and B according to the HER2 profile and the presence of proliferation genes such as CCNB1, MKI67, and MYBL2, which are generally expressed in luminal B subtype. Moreover, luminal B is characterized by a higher expression of genes connected with growth receptor signaling [30,31,32]. Based on a clinical prediction and patients’ prognosis, luminal A represents molecular subtypes with a better prognosis, low relapse, and higher overall survival rate when compared to luminal B [33]. 

Importantly, human cancer-derived cell lines, which are specific for each molecular subtype, represent powerful tools to study biological processes in cancer research because they carry specific genetic alterations of tumors they were derived from [34]. Table 1 summarizes BC subtypes associated with specific immunohistochemical signatures and the corresponding cell line used in in vitro experiments.

## 3. Chemotherapy for Breast Cancer

As mentioned above, BC is a heterogeneous disease with specific properties. The identification of BC subtypes is crucial for selecting appropriate chemotherapeutic drugs. According to the mode of action, chemotherapeutic drugs are separated into classes including antimetabolites, endocrine therapy, immunologic therapy, alkylating agents of DNA, and antimitotic drugs [4]. Antimetabolites are responsible for the induction of apoptosis during the synthesis phase. The structure of antimetabolites is analogous to that of folate, purine or pyrimidine and causes mistakes during replication. Moreover, these drugs are analogs of normal substances, which are important for normal cellular functions. Regarding enzymes that the antimetabolites suppress, they are classified into inhibitors of dehydrogenases, topoisomerases, nucleosides, and kinases [41,42]. Methotrexate is a dehydrogenase inhibitor, which acts as a competitive inhibitor of DHFR (dihydrofolate reductase) leading to accumulation of folate and subsequent inhibition of synthesis [43]. Doxorubicin represents the class of topoisomerase II inhibitors with a crucial role in the inhibition of topoisomerase II, formation of DNA adducts, and generation of oxidative stress [44]. Side effects of doxorubicin as well as other chemotherapeutical drugs are frequent complications during therapy. Decreasing the negative symptoms and higher therapeutic efficiency were allowed by the development of liposomal anthracyclines [45]. Epirubicin, similar to other anthracyclines, acts as an intercalating agent with DNA and thus interferes with transcription, resulting in suppression of RNA synthesis [46,47]. Kinase inhibitors Palbociclib and Ribociclib are both cyclin-dependent inhibitors suppressing cyclin dependent kinase (CDK) 4/6 activity to elicit the inhibition of proliferation [48]. 5-Fluorouracil, Capecitabine, and Gemcitabine are nucleoside inhibitors associated with the silencing of transcription and translation in BC [49]. Immunological therapy focuses on molecular subtypes with over-expressed HER2 receptors [50]. Herceptin and Ado-trastuzumab are two dominant agents for immunotherapy. Specifically, herceptin blocks the extracellular domains of HER2 receptor tyrosine kinase. On the contrary, Ado-trastuzumab delivers the microtubule-inhibitory agent DM1 drug into cells with an increased level of HER2. Endocrine therapy is a choice for patients with hormone-positive receptors including treatment by a synthetic analogue of anti-gonadotropin releasing hormone (Goserelin), antiprogestines (Megestrol acetate), and anti-estrogens, which are further subdivided into antagonists of ER (Tamoxifen) and an aromatase inhibitors (Trozole) [4,51]. DNA alkylating agents are substances that interact with DNA and block DNA replication. According to the fundamental mechanism of action, they are classified into 3 groups: platinum-based agents (cisplatin, carboplatin, oxaliplatin), nitrogen mustards (cyclophosphamide, chlorambucil), and organophosphorus compounds (Thiotepa) [42]. The antimitotic chemotherapeutical concept is a highly validated treatment option that reduces proliferation and invasion of cancer cells via modulation of cellular division mediated by alteration of microtubule function. Consequently, modification of microtubules leads to cell cycle arrest and the subsequent apoptosis pathway. Inhibitors of microtubules represent two groups of drugs: synthetic and natural. Ixabepilone, a semi-synthetic analog of epothilone B, belongs to synthetic chemo drugs with antimitotic properties for treatment of metastatic BC. Epothilones are defined as microtubule inhibitors, highly-effected against PTX-resistant cells [52]. Interestingly, higher effectiveness of Ixabepilone than PTX against resistant cells is caused by the different binding sites of the drug at microtubules. The category of naturally derived antimitotic drugs represents marine and plant substances interacting with tubulin. Eribulin mesylate is an antineoplastic drug belonging to the halichondrin class that interacts with microtubules, leading to anaphase/metaphase arrest [53]. Taxanes and alkaloids are chemotherapy drugs derived from plants. Taxanes (PTX, Cabazitaxel and Docetaxel) represent the most applied chemotherapy approaches and along with anthracyclines are the first line of treatment for patients with metastatic as well as early-stage BC [54]. Importantly, certain dominant-negative mutations of genes associated with the regulation of mitosis are responsible for the development of resistance of cancer cells, and thus the effectiveness of taxane therapy is decreased [55]. The second group of natural plant-derived chemo-drugs are alkaloids such as Vinblastine that acts as microtubule-disruptive agents inhibiting tubulin polymerization [56]. In summary, there are numerous chemotherapeutics against BC with different mechanisms of action. The choice of appropriate therapy is critical for patients, and only the precise determination of the tumor intrinsic type and stage of diseases can decrease the recurrence or metastasis development.

## 4. Paclitaxel: Fundamental Drug in Chemotherapy and Novel Advances in its Application

As noted above, antimitotic chemotherapeutics suppress the polymerization dynamic of microtubules, resulting in the induction of mitotic arrests as a consequence of the activation of the mitotic checkpoint. Consequently, PTX, a member of taxanes, represents one of the most important antineoplastic drugs frequently used in the treatment of numerous types of cancers including BC.

### 4.1. The Origin of Paclitaxel 

PTX, trademarked by Bristol-Myers Squibb (BMS) as Taxol™ in 1992 [57], is an antimitotic, anticancer drug that was approved by the FDA (Food and Drug Administration) in 1994 for use in BC [58]. PTX is clinically used to treat solid tumors such as ovarian cancer, hormone refractory prostate cancer, and non-small cell lung cancer [59]. The active ingredient was first isolated from the Pacific Yew tree (*Taxus brevifolia)* by Mansukh Wani and Monroe Wall [60]. The drug went through additional clinical trials and testing on mouse tumor models before it was FDA approved for ovarian cancer in 1992 [57]. Due to the high demand for the drug, the slow-growing *T. brevifolia* tree was not able to provide the market and research’s needs; therefore, it was concluded that the production of PTX from *T. brevifolia* was impractical, non-environmentally conscious, and financially burdening according to the National Cancer Institute (NCI) [61,62]. In 1993, a new method of mass-producing PTX was associated with a fungus isolated from the phloem of *T. brevifolia* [63]. In 1994, a successful semi-synthetic approach of synthesizing PTX was formulated and approved by the FDA, which is the method of production until today [58,62]. 

### 4.2. Paclitaxel’s Mechanism of Action 

(I) Paclitaxel as a Polymerization Factor 

PTX binds to microtubules instead of tubulin dimers and stabilizes microtubules (polymerization) by promoting the assembly of alpha and beta tubulin subunits, the building blocks of microtubules [57,64]. The drug reduces the critical concentration of tubulin required for its assembly, therefore promoting the lengthening of the tubulin polymer [65]. The stability of the microtubules interferes with microtubules´ dynamics. Subsequently, the cell’s ability to divide is disrupted due to insufficient requirements of the mitotic checkpoint; therefore, cell division halts at the G2 or M phase. The polymerized and stable microtubules remain largely unaffected even by cold temperatures and calcium. The presence of calcium reduces PTX’s affinity for tubulin; therefore, the equilibrium of polymerization/depolymerization shifts towards polymerization to offset this effect [57,66,67]. Moreover, chondrocytes show that PTX causes cytoskeletal abnormalities in which microtubules become stubby and straight in the cytoplasm, with rough endoplasmic reticulum as opposed to fine, sinous filaments in the control group. These changes persist for 48 h after the removal of PTX. The changed microtubules dislodge ribosomes off the rough endoplasmic reticulum and fuse nearby endoplasmic reticulum complexes together [66]. PTX polymerizes only free microtubules not attached to or preexisting in the microtubule organizing centers (MTOC). Attached microtubules disappear in the presence of PTX [68]. PTX interferes with the dynamics of microtubules and microtubule polymerization and delays the progression of mitosis by inducing failure in chromosomal segregation, all of which eventually lead to the induction of apoptosis and mitotic arrest [69,70,71].

(II) Paclitaxel’s Effect Depends on Concentration

The mechanism of PTX cytotoxicity highly depends on the concentration of the drug in the cell as demonstrated in in vitro studies. Giannakakou et al. [72] documented that the reduction of proliferation of the lung carcinoma cell line A549 as well as breast MCF-7 cells after treatment by PTX at concentrations above 12 nM resulted in G2/M arrest. Interestingly, lower concentrations of PTX (3–6 nM) exerted similar potential to suppress the proliferation of cancer cells, resulting in programmed cell death [72]. A study focusing on drug concentration analyzed the effect of low doses of PTX (10 nM) on cancer cell invasiveness. In this in vitro study, researchers evaluated the impact of the non-anti-mitotic concentration of PTX resulting in the reduction of transwell invasion of MDA-MB-231 as a consequence of the regulation of voltage-dependent sodium channel expression [73]. Additionally, the potential role of low doses of PTX (20 nM) combined with a Wnt signaling inhibitor regulated the molecular events, including E-cadherin upregulation and β-catenin reduction, leading to suppression of tumor growth, metastasis, and angiogenesis in BC [74].

(III) Paclitaxel Affects Phosphorylation of Bcl-2 

Although many researchers agree that the cytotoxicity of PTX lies in its ability to cause Bcl-2 hyperphosphorylation, many other studies report dephosphorylation of Bcl-2 coinciding with apoptosis [75,76,77]. However, since apoptosis does not occur immediately after exposure to PTX, the duration of exposure and constant Bcl-2 phosphorylation contribute to the drug’s cytotoxicity [78]. On the other hand, different studies proved that phosphorylated Bcl-2 does not dimerize with BAX; therefore, it is argued that the unassociated BAX favors apoptosis [79,80]. However, the later studies are older and probably less updated than the former studies, and these conclusions are based on prostate and leukemia cell lines. 

(IV) Paclitaxel Affects Calcium Signaling 

PTX induces the depletion of calcium ions from the mitochondrial reserve through the mitochondrial permeability transition pore (PTP). The leaving calcium induces PTP to release the apoptogenic factor cytochrome C (cyto C) into the cytosol from the mitochondria to initiate apoptosis [81,82,83]. It is believed that side effects of antimitotic drugs are related to the role of those drugs in the calcium signal cascade. The severity and heterogeneity of these side effects are attributed to the alterations in mitochondrial calcium uptake in different cells. Using extremely high doses of PTX results in the rupture of the mitochondria, the release of cyto C, and the initiation of apoptosis without the efflux of calcium [84]. Reasons why peripheral neurons are primarily affected by PTX are not known, even though most cells have mitochondria. On the other hand, PTX induces apoptosis in the presence of an extracellular calcium reservoir via calcium influx when the drug is administered in high doses. However, low doses of PTX demonstrated patterns of apoptosis independent of the extracellular concentration of calcium [10].

(V) The impact of Paclitaxel on microRNA Expression Profiles

MiRNA, small non-coding RNA with a regulatory function in gene expression, can be regulated by various antineoplastic drugs including PTX. Several studies focusing on miRNA expression demonstrated cross-connection between an application of the drug and alterations in miRNA expression profiles. After PTX intervention, expression levels of let-7a and miR-205 with tumor suppressor potential targeting K-Ras and HER3 were changed in the BC cell line BT-474 [85]. Interestingly, metronomic treatment (low dose LDM) by PTX reduced the level of let-7f, while the expression of thrombospondin-1 (TSP-1) associated with anti-angiogenic potential was increased in PTX LDM therapy [86]. In summary, preclinical trials demonstrated the modulatory potential of PTX in the regulation of miRNA expression, but further research is needed for a better implication of the drug in BC treatment.

(VI) The Immunomodulatory Effects of Paclitaxel 

**Figure 1:**
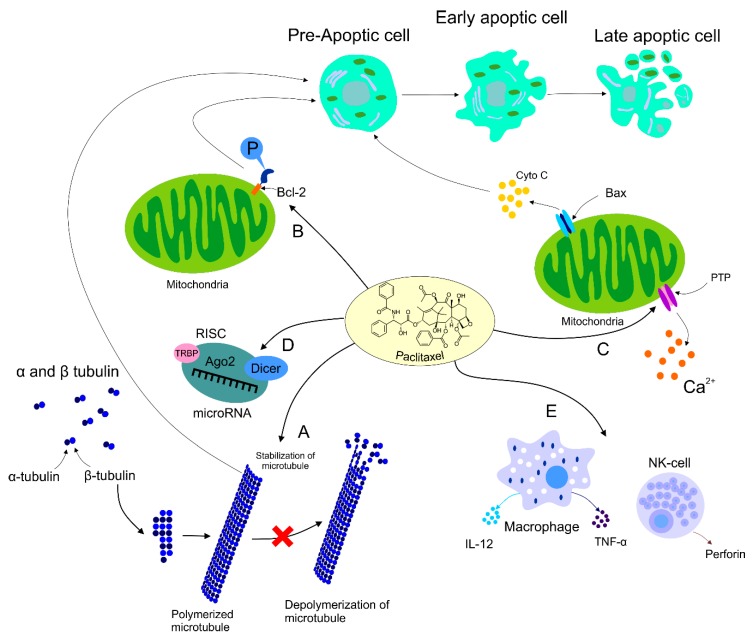
Mechanism of action of PTX. Anti-tumor mechanism of action of PTX leading to stabilization of microtubule, cell arrest, and subsequent apoptosis (A). PTX also causes activation of the immune response contributing to tumor eradication (B). The ability of PTX to inactivate Bcl-2 via phosphorylation of the anti-apoptotic protein resulting in apoptosis (C). Participation of PTX in the regulation of certain miRNAs associated with the modulation of tumor progression (D). Regulation of calcium signaling by PTX results in PTX-induced release of cyto C from the mitochondria and programmed cell death.

The role of PTX was also documented in the area of immunomodulation, with both stimulation and suppression of immune cells associated with tumor growth. On the other hand, the suppression of immune cells could have a negative impact on the host immune response against cancer development [87]. Increasing evidence supports participation of PTX in the regulation of host immunity via stimulation of macrophages leading to cytokine secretion including TNF-α or IL-12 that induce activation of natural killers (NK), dendric cells (DC), and cytotoxic T lymphocytes resulting in the eradication of tumor cells [11,88]. Additionally, direct-acting PTX was evaluated in DC via binding to Toll-like receptor localized on the DC surface, thus promoting maturation of antigen-presenting cells [89]. Furthermore, the dose-dependent administration of PTX led to an increased level of MHC class II [90]. The impact of PTX on immunomodulation was also identified in NK cells. A higher level of cytotoxicity correlated with an increased level of perforin representing the crucial effector protein of NK activity, resulting in the premise that PTX enhanced NK cytotoxicity in a dose-dependent manner [91]. Further research focusing on the regulation of the immune response in cancer is necessary for better understanding of an association between effects of PTX and immunopharmacology. The mechanisms of PTX in antineoplastic processes described above are summarized in Figure 1. 

### 4.3. Paclitaxel’s Effect on HER2+ Breast Cancer

The HER2+ subset of BC is more actively biochemically studied as opposed to other subsets. The efficacy of PTX in HER2+ BC patients was inconsistent and contradicting. In 1998, HER2+ BC was found to be biochemically resistant to PTX. The over-expression of HER2 upregulates p21cip1, which inhibits p34cdc2 that is normally activated by PTX in order to induce apoptosis of cancerous cells at the G2/M phase; therefore, the cytotoxic effect of PTX was inhibited [92]. More recent studies conducted on 3121 node-positive postoperative patients showed that the addition of PTX in adjunction to doxorubicin plus cyclophosphamide decreased the rate of recurrence and death significantly upon 10-year follow up [93]. In contrast, out of 46.7% patients responding to taxanes, 65.2% represented HER2+ and 35.5% HER2− tumors [94]. The author suggested that PTX works on a signaling transduction pathway specific to HER2+ cancer. PTX activates the tumor suppressant protein p35 and the CDK inhibitor p21WAF1. On the other hand, p21WAF1 is not activated due to the usual activation of p35 since cancer cells lack the presence of this tumor suppressor. Nevertheless, it is suggested that p21WAF1 activation is c-raf-1 dependent since PTX activates c-raf-1, and the accumulation of p21WAF1would not be possible in the case of c-raf-1 depletion [94,95].

### 4.4. Dose Ranges Administered

A number of studies evaluated different doses of PTX [96]. Table 2 demonstrates the established dose ranges clinically administered to BC patients according to the state and diagnosis of the patient.

Patients pre-treated with anthracyclines did not exhibit a statistically significant response rate compared to those who were not pre-treated with anthracyclines [100]. Using taxanes in conjunction with anthracyclines lowers the risk of disease recurrence and relapse [98]. Although Table 2 shows the clinical dose ranges, a healthcare provider might administer different doses on a different schedule upon clinical assessment. Additionally, Table 3 demonstrates the efficacy of PTX when used in combination with other drugs. As noted, the medical literature does not provide the immunohistochemical or histological profile of the BC, leading to the suggestion that the differential response could be due to the versatile patient body in the trials. 

### 4.5. Breast Tumor Resistance to Paclitaxel

Chemoresistance is a major problem of cancer treatment associated with poor response, tumor recurrence, and metastases and represents the leading cause of mortality in BC patients [106,107]. Primary chemoresistance refers to resistance occurring prior to treatment. On the other hand, acquired resistance may develop over time after the chemotherapeutic exposure [107]. Accordingly, treatment with PTX might increase acquired resistance, which leads to chemotherapy failure [108]. Importantly, the mechanisms associated with the challenging and complex nature of chemoresistance [107] are still not clear [109]. 

Firstly, over-expression of efflux drug proteins is associated with resistance to more than one class of chemotherapeutic agents. The ATP-binding cassette (ABC) superfamily of drug efflux proteins includes P-glycoprotein (P-gp) also known as ABCB1 or MDR-1 [107]. The ABCB gene is involved in the resistance to PTX mediated by over-expression of P-gp, which is consequently associated with the efflux of the drug outside of cells [110]. Importantly, the sensitivity to PTX in PTX-resistant sublines of SK-BR-3 and MCF-7 cells increased significantly but not completely through silencing of ABCB1. Therefore, multiple mechanisms are suggested to be included in PTX resistance in BC cells [109]. 

Moreover, PTX resistance is associated with spindle assembly checkpoint (SAC) due to the importance of the checkpoint function in PTX sensitivity. Therefore, SAC proteins including Mad2, BubR1 or Aurora A are potentially important markers of PTX resistance [8]. However, suppression of Mad2 and BubR1 in PTX-treated cells eliminated the checkpoint function, which led to the PTX resistance correlating with the reduction of the cyclin-dependent kinase-1 activity [13]. Moreover, over-expression of Aurora kinase A (Aur-A) and FOXM1 was observed in PTX-resistant TNBC cells, suggesting the role of Aur-A in the protection of tumor cells against PTX [111]. Similarly, aberrantly regulated expression of FOXM1 and KIF20A was associated with PTX resistance in MCF-7 cells [112]. 

Furthermore, alterations in the expression of microtubule-associated proteins (MAPs), such as Tau or MAP4, are also important markers of PTX sensitivity [8]. Microtubule-associated protein Tau may be used as a marker for selection of patients for PTX therapy, as its low expression makes microtubules more vulnerable and BC cells more sensitive to PTX [113].

Moreover, PTX resistance represents an important area of molecular regulation mechanisms connected with changes in miRNA expression that plays a crucial role in chemoresistance to numerous therapy approaches [102,114]. The acquisition of resistance to PTX was identified in an experimental study focusing on the expression of miR-200c-3p. Analyzed miRNA was directly associated with the regulation of SOX2, and over-expression of miR-200c-3p contributed to resistance of BC cells to PTX therapy [14]. Similarly, miR-107 targeted genes related to resistance to Taxol treatment. Upregulation of miR-107 correlated with a decrease in the expression of the oncogene TRIAP1 [115]. Moreover, experimental analysis of MDA-MB-231 clearly proved the inverse correlation between miR-16, which is characterized as a tumor-suppressor element regulating anti-neoplastic events in the cell, and IKBKB. In summary, increased expression of IKBKB corresponded to the chemo-drug resistance to PTX [116]. Furthermore, the participation of over-expressed Lin28 that essentially induced an increase in the expression of Rb and p21 as well as a decrease in the level of let-7 resulted in drug-resistance to Taxol [117]. In addition, recent evidence suggested an interaction between the downregulation of miR-22 and the progression of BC. Using real-time PCR, the lower level of analyzed miR-22 targeting the NRAS oncogene was found to correlate with the reduction of the cancer cells’ sensitivity to PTX [118].

Additionally, increased expression of the actin-binding protein (CapG) promoted PTX resistance in BC cells and xenograft models and was related to the PTX resistance in BC patients through targeting CapG-mediated hyperactivation of the PI3K/Akt pathway [106]. Moreover, increased Y-box protein (YB)-1 levels and decreased EGR1 (early growth response protein 1) mRNA levels correlated with PTX resistance in MDA-MB-231, while a proposed mechanism suggested the biological link between EGR1 and YB-1 and that an increase in YB-1 decreased EGR1 [119]. Interestingly, post-translational modifications such as sumoylation modulated sensitivity to PTX in the BC cell line MCF-7. Data suggested an important role of the post-translational mechanism in the regulation of FOXK2, directly linked to drug resistance in in vitro experiments. Consequently, sumoylation functions as a positive regulator of FOXK2 and its transcriptional activity that subsequently enhances the cytotoxic response to PTX [120]. 

Taken together, the above-discussed data suggested an important role of various mechanisms associated with ABC, SAC or MAP proteins [107] as well as epigenetic modulation [120] in numerous cellular processes associated with the chemoresistance to PTX. In conclusion, in order to improve the use of chemotherapeutic agents, such as PTX, it is important to clarify the mechanisms underlying the issue of BC chemoresistance [107].

### 4.6. Paclitaxel’s Side Effects

Importantly, PTX is a chemotherapeutic agent arresting mitosis via microtubule stabilization and consequent induction of apoptosis that is widely used to treat BC. However, the effectiveness of PTX is limited by various side effects associated with its use [121,122].

Major side effects of PTX are hypersensitivity and neuropathies. Due to its poor solubility, PTX has to be formulated in a lipid-based solvent polyoxyl castor oil, also known as Cremophor™, and dehydrated ethanol. However, this vehicle is associated with histamine-mediated hypersensitivity reactions, sensory neuropathy, or impairment of drug delivery as well as limitation of its effectiveness [123,124,125]. PTX hypersensitivity including dyspnea, bronchospasm, urticaria, flushing, erythematous rash, hypotension, angioedema, chest pain, abdominal pain, fever, or rigors is usually visible within the first ten minutes of drug administration. Moreover, PTX hypersensitivity usually results in the immediate halt of the treatment. However, premedication with dexamethasone, diphenhydramine, or cimetidine can reduce hypersensitive reactions. Importantly, reducing the PTX dose by 20% or administration of Amitriptyline may alleviate some of the neuropathic side effects [126]. As mentioned above, peripheral neuropathy is associated with high doses of numerous chemotherapeutic drugs including PTX. In this regard, Vahdat et al. [127] analyzed the reduction of neuropathy after high-dose PTX with oral administration of 10 g glutamine (starting 24 hours after PTX completion), 3 times a day over 4 days. Their clinical data suggested that patients treated with glutamine had significantly decreased levels of severity connected to peripheral neuropathy compared to the placebo control group [127]. Nevertheless, the severity of neuropathy noted in patients depends on the time of the PTX dosage repetition. Patients with weekly administration of PTX showed signs of grade 2, 3, and 4 neuropathy more frequently than patients who were subjected to the drug every three weeks. In addition, patients who received PTX complained of neutropenia, febrile neutropenia, and infection, although the most common side effect was neuropathy [98]. Although the exact mechanism of PTX causing neuropathy is unknown, the administration of Ethosuximide, an anti-epileptic drug and a selective T-type calcium channel blocker, completely reversed the neuropathic symptoms, which suggests that T-type calcium channels are ingrained in the mechanism [128]. The administration of G-CSF (granulocyte colony-stimulating factor) with PTX significantly decreased the severity of neutropenia [126]. Additionally, the establishment of polar micelles of PTX in the plasma compartment causes non-linear pharmacokinetics and drug entrapment. Consequently, the pharmacodynamics of the drug changes, which may cause a risk of systemic toxicities attributed to the substantial increase in its systemic exposure and reduction of its systemic clearance [125].

Moreover, Cremophor™ was also suggested to alter pharamacokinetics of other anticancer drugs, e.g., anthracyclines [129]. Despite premedications with antihistamines and corticosterois to reduce hypersensitivity reactions, this formulation also requires a special infusion set in order to minimize exposure to releasing diphtalates and prolonged infusion time [125]. 

Additionally, administration of PTX has been associated with cardiotoxicities such as bradyarrythmias, tachyarrythmias, atrioventricular and bundle branch blocks, and cardiac ischemia [130]. However, when combined with doxorubicin, congestive heart failure surfaced [99]. The cardiotoxic effects of PTX are thought to be the result of Cremophor™ hyperstimulation of the histamine receptors in cardiac cells due to the huge influx of histamine in the presence of Cremophor™ [130,131]. 

### 4.7. Albumin-Bound Paclitaxel and Comparison with Its Conventional Alternative

**Figure 2:**
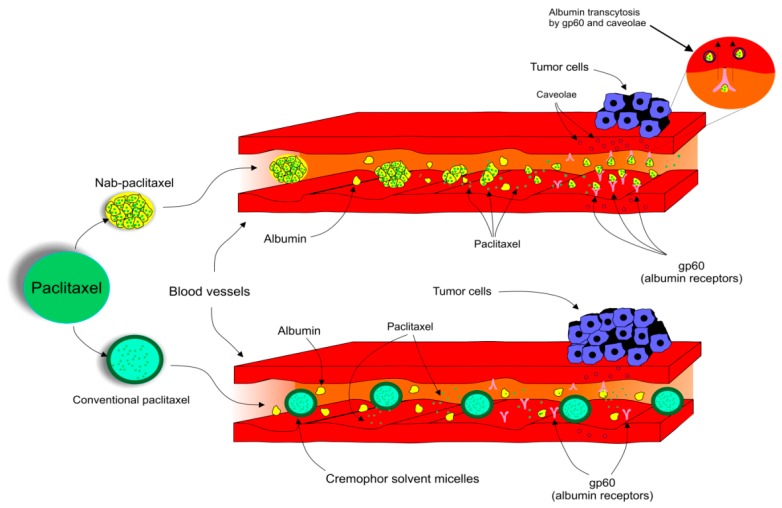
Transcytosis, a receptor-mediated transport of nab-PTX into tumor cells.

Due to the adverse reactions associated with conventional PTX, a need to develop another way it is used is urgently required [123,124,125]. The pharmacokinetics and pharmacodynamics of PTX have been improved by the development of a nanocarrier delivery system. Nab-PTX, which is defined as PTX bound to albumin nanoparticles [132], is a solvent-free preparation allowing PTX to be delivered as a suspension of albumin nanoparticles [133]. Nab-PTX was designed with an aim to improve the therapeutic potential of PTX and to reduce its toxicity and side effects [134]. Importantly, the advantage of nab-PTX is an ability to increase the delivery of albumin to tumors through receptor-mediated transport, also known as transcytosis (shown in Figure 2) [135,136]. Cav-1 is the main component of plasma membrane invaginations (calveolae), and deregulation of tumor Cav-1 is highly implicated in BC. Moreover, loss of stromal Cav-1 plays an important role in disease recurrence and overall worse prognosis of BC patients [137]. Due to the presence of albumin, the binding of nab-PTX to gp60, an albumin receptor on endothelial cells, leads to the activation of caveolin-1 (Cav-1) and formation of calveolae [135]. Consequently, calveolae transport nab-PTX through endothel into the extracellular space including the tumor interstitium [135]. SPARC (secreted protein acidic and rich in cysteine) is defined as an albumin-binding glycoprotein that is frequently over-expressed in cancer cells [138]. Therefore, in the tumor intestitium, SPARC binds to albumin-bound PTX, which facilitates release of PTX near cancer cells and increases the antitumor efficacy of nab-PTX [135]. Nab-PTX has been proven to be 33% more effective than conventional PTX in MX-1 tumor xenografts due to its interaction with SPARC that is over-expressed especially in BC [139]. In addition, nab-PTX does not require premedication, special IV tubing, or a long perfusion time [140].

A number of clinical trials evaluated the impact of nab-PTX on therapeutic efficacy as well as its association with side effects. Nab-PTX was related to fewer side effects in comparison with conventional PTX, as a lack of hypersensitivity, due to the absence of the Cremophor™ diluent [141]. Weekly neoadjuvant administration of nab-PTX at 100 mg/m^2^ was associated with good response and tolerability in patients with stage II to IV BC [142]. Additionally, a phase II study of nab-PTX administered weekly in patients with metastatic BC heavily pretreated with taxanes revealed that both 100 mg/m^2^/weekly and 125 mg/m^2^/weekly demonstrated the same antitumor activity. However, there were no severe hypersensitivity reactions, and patients with treatment-limiting peripheral neuropathy could be restarted on a reduced dose of nab-PTX after a few weeks delay [140]. Additionally, neoadjuvant therapy with weekly nab-PTX in luminal early BC patients indicated significant drug antitumor activity indicated via a residual cancer burden rate of 0 + 1 with low rates of grade 3-4 toxicity [143]. Moreover, nab-PTX at a dose of 175 mg/m^2^/3 weeks was associated with less frequent chemotherapy-induced peripheral neuropathy in HER2- metastatic BC patients [144]. A multicenter phase II trial of ABI-007, the first biologically interactive albumin-bound PTX in a nanometer-sized particle, in women with metastatic BC revealed significant anticancer activity, allowing safe administration of high ABI-007 doses without premedication. However, toxicities typical of PTX, including neutropenia (grade 4, 24%), sensory neuropathy (grade 3, 11%), and febrile neutropenia (grade 4, 5%), were observed [145]. 

Importantly, there is interesting evidence evaluating a comparison of the effectiveness of conventional and nab-PTX. A study using data from an electronic medical record database across the USA revealed that nab-PTX improved clinical effectiveness, demonstrated by a longer time to treatment discontinuation and the time to the next treatment of metastatic BC patients when compared with conventional PTX [16]. Moreover, ABI-007 demonstrated a higher efficacy and safety profile when compared with conventional PTX in women with metastatic BC. Despite that grade 3 neuropathy was more frequent in the ABI-007 group, it was easily manageable (also no hypersensitivity, and grade 4 neutropenia was significantly lower for ABI-007 compared to PTX) [146]. However, Gianni et al. [147] evaluated the ability of nab-PTX to improve the outcomes of early and locally advanced HER2− BC in comparison with PTX in a neoadjuvant setting. Consequently, improvement of pathological complete remission after nab-PTX was not statistically significant [147]. 

## 5. Novel Insights into the Application of Paclitaxel in Clinical Practice

Due to the disadvantages of conventional PTX, which include limited solubility and side effects or localization to non-tumor areas, it is necessary to develop more effective ways of cancer cell targeting and delivery of PTX [148,149]. As was demonstrated by using nab-PTX in BC treatment, a nanocarrier system is an effective way to target cancer cells as well as to minimize side effects associated with conventional PTX. Recently, significant progress has been observed concerning the carrier systems developed to enhance PTX effectiveness. Importantly, the use of phytochemicals in cancer therapy is currently becoming highly attractive and advantageous. Therefore, natural plant compounds appear to possess beneficiary effects in the increase of the efficacy and reduction of toxicity associated with PTX [150,151].

HPA (heparanase) aptamer functionalized PTX-encapsulated PEGylated PLGA (poly(lactic-co-glycolic acid)) nanoparticles were designed to target BC cells via targeting HPA, which is highly expressed in cancer cells including TNBC [152]. Moreover, PTX encapsulated in glutathione (GSH)-sensitive amphiphilic hyperbranched poly (amide-amine) (mPEG-PLGA-HPAA) micelles was analyzed in order to determine the ability to enhance its chemotherapeutic potential in MDA-MB-231 cells. Consequently, the drug carrier led to improvements of PTX efficacy and exerted great biocompatibility both in vitro and in vivo [153]. Furthermore, PTX-loaded folate-coated long-circulating and pH-sensitive liposomes (SpHL-folate-PTX) promoted cellular uptake and anticancer activity in vitro as well in vivo [154]. Moreover, encapsulation of PTX into tannic-acid nanoparticles (TAP NPs) exerted a superior antitumor effect when compared with conventional PTX [155]. Similarly, a multifunctional folate-conjugated curcumin and PTX-loaded lipid nanoparticle enhanced folate-targeted delivery of the drug and inhibition of multidrug resistance in tumor cells [156]. Moreover, PTX chemoresistant MDA-MB-231 cells were sensitized via sequential release of PTX and epigallocatechin gallate from PLGA-casein core/shell nanoparticles [157]. Importantly, co-encapsulation of PTX with naringin in mixed polymeric micelles was designed in order to enhance the anticancer activity of PTX against BC cells [158]. Additionally, a mixed polymeric micelle used for co-delivery of PTX and retinoic acid may promote the therapeutic efficacy of PTX and reduce its side effects [159]. 

As was indicated by the above-mentioned recent studies, PTX delivery mediated through the use of nanocarriers represents great progress in cancer treatment by chemotherapeutic agents. Moreover, improvements in the technologies using the advantages of natural plant compounds to increase the efficacy of PTX indicate potential to maximize therapeutic effects and minimize side effects of PTX in cancer treatment [150,151,153].

## 6. Conclusions

BC represents the most frequent malignancy as well as the main cause of cancer-related deaths in women [2]. The choice of appropriate therapy is a highly important factor concerning the possibilities of patients for successful treatment. PTX is an antimitotic chemotherapeutic agent widely used in cancer treatment [57]. Despite the possibility to isolate the drug from the Pacific Yew tree [60], the high demand for PTX led to its semi-synthetic production [58,62]. PTX affects human cells via control of microtubule polymerization [57,64], Bcl-2 phosphorylation [75,76,77], mitochondrial calcium efflux and influx [81,82,83], and modulation of miRNA expression profiles [85,86]. Moreover, current experimental data suggests a direct association between PTX therapy and an impact on the immune system during carcinogenesis [87,88,89,91]. PTX is an effective adjuvant drug in BC that is most effective in the PTX + trastuzumab combination due to its high disease-free survival and 3-year recurrence-free survival rates in addition to a low frequency of reported adverse effects and no cardiological toxicities [104]. Nevertheless, hypersensitivity and other side effects remain a major downside to the use of PTX, mainly due to the Cremophor™ diluents [123,124,125], which can be mitigated by the alternate use of nab-PTX in which fewer hypersensitivity side effects are reported [135,136]. Therefore, nab-PTX is safely infused at higher doses in BC when compared with conventional PTX, resulting in a shorter time of infusion. Moreover, nab-PTX is also associated with higher response rates and no need for premedication [160]. Consequently, the success in creating nab-PTX represents the potential to create an anti-cancer drug that has relatively mild side effects [132].

Furthermore, chemotherapy resistance represents a serious problem in cancer treatment. Evidence shows that PTX resistance is mediated via several mechanisms involving, e.g., members of the ABC superfamily of drug efflux proteins [107] or MAP proteins as well as SACs [8]. Resistance to PTX may also be modulated through other mechanisms, including epigenetic regulation connected with miRNA [102,114] or posttranslational modifications [120].

Above all, from the beginning of its use to treat BC in 1994 [57,58,59], PTX has undergone significant development. However, PTX, due to its role in the targeting of microtubules, structures found in all eukaryotic cells, is not tumor specific. Nevertheless, mitotic cells are most sensitive to PTX [161,162]. A perspective option to increase the specificity of PTX in the recognition of BC cells seems to be the application of nanocarriers conjugated with particles that target receptors over-expressed in BC cells [163]. Importantly, the advantages of the development of novel strategies to enhance the efficacy of PTX include the targetability, toxicity, and selectivity toward cancer cells [148].

In conclusion, PTX is a fundamental drug used in BC treatment. However, due to discrepancies associated with its use in the clinical sphere, it is highly important to improve its properties in association with the reduction of cancer cell resistance to PTX, increase its effectiveness in cancer therapy, and reduce the side effects related to the use of conventional PTX. The use of new alternatives of conventional PTX is based on the regulation of molecular pathways and epigenetic mechanisms, which currently represent a highly topical field of scientific research in BC patient management. Above all, further progress in the development of new carriers for targeting cancer cells, as well as the use of phytochemicals in this field, represents a challenge to be overcome by cancer research.

Some of the limitations of this study lie in the non-specific designation of BC subtypes in the afore-mentioned clinical trials, as no immunohistochemical profile was specified in the eligibility criteria. Very few studies focused on PTX’s mechanistic approach in BC subtypes other than HER2+, which limited the scope of this study.

## Figures and Tables

**Table 1:** Intrinsic types of BC with corresponding cell lines.

BC type	BC subtype [35]	Immunohistochemical profile [36]	Cancer cell line [35]
Luminal	Luminal A	ER+, PR+, HER2−, Ki67 low expression	BT483, CAMA1, HCC712, EFM19, HCC1428, HCC712, IBEP2, KPL1, LY2, MCF7, MDAMB134, MDAMB134VI, MDAMB175, MDAMB175VII, MDAMB415, T47D, ZR751, ZR75B
	Luminal B (Luminal-HER2+)	ER+, HER2+, PR−, or Ki67 high expression	BSMZ, BT474, EFM192A, MDAMB330, MDAMB361, UACC812, ZR7527, ZR7530
TNBC	Basal-like	ER−, PR−, HER2−	BT20, CAL148, DU4475, EMG3, HCC1143, HCC1187, HCC1599, HCC1806, HCC1937, HCC2157, HCC3153, HCC70, HMT3522, KPL-3C, MA11, MDAMB435, MDAMB436, MDAMB468, MFM223, SUM185PE, SUM229PE
	Claudin-low [37,38,39]	ER−, PR−, HER2−, claudin 3−, claudin 4−, claudin 7− and E-cadherin [40]	BT549, CAL120, CAL51, CAL851, HCC1395, HCC1739, HCC38, HDQ-P1, Hs578T, MDAMB157, MDAMB231, SKBR7, SUM102PT, SUM1315M02, SUM149PT, SUM159PT
Non-hormonal related HER2+	HER2	ER−, PR−, HER2+ over-expression	AU565, HCC1008, HCC1569, HCC1954, HCC202, HCC2218, HH315, HH375, KPL-4, MDAMB453, OCUB-F, SKBR3, SKBR5, SUM190PT, SUM225CWN, UACC893

Abbreviations: BC, breast cancer; ER, estrogen receptor; HER2+, human epidermal growth factor receptor 2 positive; HER2−, human epidermal growth factor receptor 2 negative; PR, progesterone receptor, TNBC, triple-negative breast cancer.

**Table 2:** The administered amount of PTX in correspondence with the patient’s condition and diagnosis.

Condition	Administration Schedule	Concentration Range	Reference
Adjuvant therapy with doxorubicin (node-positive or high-risk node-negative BC)	Every 3 weeks	175 mg/m^2^ IV perfusion over 3 h (4 courses)	[97]
	Weekly	80 mg/m^2^ IV perfusion over 1 h (12 courses)	[98]
Failure of neoadjuvant therapy (MBC or relapse within 6 months of neoadjuvant therapy)	Every 3 weeks	175 mg/m^2^ IV perfusion over 3 h	[97]
Untreated MBC	Every 3 weeks (max. of 8 cycles)	200 mg/m^2^ IV infusion over 3 h + total dose of 480 mg/m^2^ doxorubicin 25 mg oral prednisone pre-treatment (12 h before treatment) 10 mg intramuscular chlorpheniramine + 300 mg intravenous cimetidine (both 30 min before PTX)	[99]

Explanatory notes: +, and/in combination with; h, hours; min, minutes. Abbreviations: BC, breast cancer; MBC, metastatic breast cancer; PTX, Paclitaxel.

**Table 3:** Efficacy of PTX as an adjuvant therapy.

Neoadjuvant Drug Combination	Patient Eligibility	Concentration Range	Efficacy	Reference
PTX after Doxorubicin + Cyclophosphamide	Node-positive BC with resected adenocarcinoma	60 mg/m^2^ doxorubicin + 600 mg/m^2^ cyclophosphamide (IV infusion for 30 min to 2 h every 21 days, −4 cycles +4 cycles of 225 mg/m^2^ PTX (day 1 of each cycle)	PTX + doxorubicin + cyclophosphamide: ↑ DFS by 17% Acceptable toxicity	[101]
PTX + Bevacizumab	MBC patients with/without previous hormonal therapy or adjuvant chemotherapy	90 mg/m^2^ PTX (day 1, 8, 15 every 4 weeks) + 10 mg/kg (day 1 and 15)	↑ progression-free survival (in comparison to PTX alone) ↑ frequency of hypertension, proteinuria, headache, cerebrospinal ischemia	[102]
PTX + Ttrastuzumab	Breast adenocarcinoma patients (tumor no larger than 3 cm, node-negative, min. LVEF of 50%, adequate hematopoietic and liver function)	80 mg/m^2^ PTX for 12 weeks + 4 mg/kg trastuzumab (day 1) → 2 mg/kg weekly (12 doses)	98.7% disease-free survival 99.2% 3-year rate of recurrence-free survival (95%CI) 2.92% of patients reported adverse effects	[103]
PTX + Trastuzumab then post-operative Doxorubicin + Cyclophosphamide	Stage II or III BC patients	Dexamethasone pretreatment (20 mg) + diphenhydramine (12 and 6 h before treatment) and H2-blocker (50 mg) Trastuzumab (one-time loading dose 4 mg/kg) → weekly 2 mg/kg IV infusion for 11 weeks + 175 mg/m^2^ of IV PTX over 3 h (every 3 weeks, 4 cycles) 2–5 weeks post-op: doxorubicin + cyclophosphamide	75% clinical response with 18% complete pathologic response Stage 3 tumors responded more than stage 2 tumors	[104]
PTX + rhG-CSF	BC patients (last radiation therapy at least 4 weeks prior to chemotherapy)	250 mg/m^2^ of IV PTX (for 24 h every 21 days, dose adjusted to granulocyte and platelet nadirs) 5 μg/kg/d of rhG-CSF (subcutaneously on day 3 through 10/cycle)	CR—12% of patients PR—50% of patients Inverse correlation between response and median age of patients Minimal toxic effects	[105]

Explanatory notes: + plus/and; → followed by; ↑ increase. Abbreviations: BC, breast cancer; CR, complete response; DFS, disease-free survival; LVEF, left ventricular ejection fraction; MBC, metastatic breast cancer; PR, partial response; PTX, Paclitaxel; rhG-CSF, recombinant human granulocyte colony-stimulating factor; h, hours; max., maximum; min., minimum.
